# Perspectives on goal setting: Video‐reflexive ethnography with speech–language therapists and clients

**DOI:** 10.1111/1460-6984.13138

**Published:** 2024-12-05

**Authors:** Laurien Brauner, Karin Neijenhuis, Ruth Dalemans, Philip J. van der Wees, Ellen Gerrits

**Affiliations:** ^1^ Research Centre Healthy and Sustainable Living HU University of Applied Sciences Utrecht Utrecht the Netherlands; ^2^ Department of Languages, Literature and Communication, Institute for Language Sciences (ILS) Utrecht University Utrecht the Netherlands; ^3^ Research Centre Innovations in Care Rotterdam University of Applied Sciences Rotterdam the Netherlands; ^4^ Department of Health and Welfare Zuyd University of Applied Sciences Heerlen the Netherlands; ^5^ IQ Health and Department of Rehabilitation Radboud University Medical Center Nijmegen the Netherlands

**Keywords:** person‐centred care, shared goal setting, speech–language therapy, video‐reflexive ethnography

## Abstract

**Background:**

Goal setting is an essential step in the clinical reasoning process of speech and language therapists (SLTs) who provide care for children, adolescents and adults with communication disorders. In the light of person‐centred care, shared or collaborative goal setting between the SLT and client is advised in (inter)national guidelines. SLTs face challenges in implementing (shared) goal setting as theoretical frameworks and practical interventions are scarce and less applicable to use with a wide range of communication vulnerable populations.

**Aims:**

A first step in developing theory and practical interventions is to explore first‐hand experiences of SLTs and clients about day‐to‐day goal‐setting practice. This study was guided by the following research question: What are the perspectives and needs of SLTs and persons with communication disorders regarding (shared) goal setting in routine SLT services?

**Methods & Procedures:**

The qualitative study was carried out in the setting of routine speech–language therapy services in community practices, primary education and neurological rehabilitation in the Netherlands. Data collection followed the principles of video‐reflexive ethnography, using video footage of goal‐setting conversations to facilitate semi‐structured, reflexive interviews. Data analysis was based on reflexive thematic analysis. A total of 12 interviews were conducted with client–SLT dyads, covering perspectives from children, parents and adults with a range of communication difficulties and their SLTs.

**Outcomes & Results:**

Data analysis resulted in four themes, of which two contain subthemes. Each theme represents a central organizing concept found in SLT and client interviews. The themes were identified as: (1) goal setting is a complex process; (2) goal talk needs to be communication accessible; (3) communicative participation goals are hard to grasp; and (4) the importance of relationships. Topics such as power imbalance, communication vulnerability, effective communication strategies, and motivation and trust are explored under these themes.

**Conclusions & Implications:**

SLTs are encouraged to view shared goal setting as a process that needs to be explicitly planned and communicated with clients regardless of their age or communication vulnerability. SLTs have expert knowledge and skills when it comes to supporting communication and applying these skills during goal talks might strengthen shared goal setting and foster a therapeutic relationship. There is a need to concretely conceptualize and embed shared goal setting in policy and clinical guidelines. The themes reported have tentative clinical implications for developing such policy, and shared goal‐setting interventions for SLT practice, under the condition that SLTs and people with communication disorders are continuously involved.

**WHAT THIS PAPER ADDS:**

**What is already known on the subject**

- SLTs want to set meaningful goals together with their patients but lack theory and resources to effectively shape the goal‐setting process. Few studies have directly reported on the perspectives and needs of SLTs and patients regarding goal setting. Patients generally perceive goal setting as a vague activity in which they minimally participate. SLTs want to involve patients in the goal‐setting process and describe the potential benefits, but they also want to report barriers on the systemic and professional competence level.

**What this paper adds to the existing knowledge**

- SLTs and patients perceive shared goal setting as a multifaceted process, rather than a one‐off conversation. This process holds potential vulnerabilities for SLTs and patients alike, and the themes in this study propose potentially helpful ingredients to mediate this vulnerability and shape the goal‐setting process.

**What are the potential or clinical implications of this work?**

- To take the first steps towards effective shared goal setting, SLTs should embrace the element of discovery in goal setting and apply their expert knowledge in supporting communication. To develop practical interventions for SLTs, shared goal setting needs to be further conceptualized and embedded in policy and clinical guidelines.

## INTRODUCTION

Speech and language therapists (SLTs) are encouraged to deliver person‐ and family‐centred care in numerous policies and position statements because of its potential positive outcomes for children, adolescents and adults with communication disorders (Forsgren et al., 2022). Shared or collaborative goal setting between SLT and client has been identified as an important practice in implementing person‐ and family‐centred care (Baylor & Darling‐White, 2020; Klatte et al., 2023). Setting goals in a collaborative way is imperative for setting meaningful goals that are focused on communicative participation, which is the most important outcome of speech–language therapy services (Baylor & Darling‐White, 2020). Despite these advantages, little is known about SLTs’ and clients’ perspectives and needs regarding day‐to‐day (shared) goal‐setting practice.

In clinical guidelines across communication disorders, goal setting is usually described as a step between assessment and therapy planning and refers to intentionally creating treatment goals which reflect a desired future state to be achieved as a result of SLT interventions (Kuiper & Lacroix, 2021; Taylor‐Goh, 2005; Levack et al., 2015). The concept of ‘shared’ or ‘collaborative’ goal setting in SLT lacks a general definition, although work from different client populations shows common descriptions of shared goal setting. Drawing on the work of An & Palisano (2014), Klatte et al. (2023) and Melvin et al. (2020) on family‐centred care, shared goal setting is described as an element of collaborative practice that requires a two‐way interaction style which is mutually supportive. Hersh et al. (2012) made similar conclusions about shared goal setting based on their work within the Goals in Aphasia Project. They state that shared goal setting includes understanding each other's perspectives and having real choices based on accessible information and the effort to continuously empower people and their families to participate in goal setting (Hersh et al., 2012). When seeking to understand the underlying principles important for goal setting in speech and language therapy for communication with vulnerable individuals, the focus on age or diagnosis becomes less critical. Another aspect that supports viewing shared goal setting as an age and disorder generic approach, is the notion that it leads to goals addressing communicative participation of individuals. Communicative participation is a crucial outcome of SLT intervention and is defined as ‘taking part in life situations where knowledge, information, ideas or feelings are exchanged. It may take the form of speaking, listening, reading, writing or nonverbal means of communication’ (Eadie et al., 2006, p. 4). Research shows that experienced barriers to communicative participation are similar in people with communication disorders regardless of etiology/diagnosis (Alons et al., 2024; Baylor et al., 2021; Ter Wal et al., 2023). This aligns with the International Classification of Functioning, Disability and Health (ICF) framework, which prioritizes functional abilities over diagnostic categories (WHO, 2001).

In many settings, SLTs face challenges in applying shared goal setting, because available assessment and treatment options still focus on impairment and performance of communicative tasks in the therapy room. Another challenge is the lack of guiding principles, and sufficient clinical resources that are suitable for use with people who are communication vulnerable (Baylor & Darling‐White, 2020). Communication vulnerable individuals ‘struggle to communicate effectively in specific environments or situations. They struggle to express their needs, wishes and values and/or to understand the information in conversations with professionals (Stans et al., 2018, p. 849). Specifically, communication vulnerability may be related to speech, language, hearing, vision and cognitive impairments, as well as a limited language proficiency in the majority language, limited literacy skills, little knowledge about healthcare, and/or cultural, sexual identity or religious differences (Blackstone et al., 2011).

If we want to move forward in facilitating shared goal setting it is key to have a thorough understanding of the perspectives and needs of SLTs and their clients with communication disorders. Research reporting on first‐hand experiences with day‐to‐day goal‐setting practice in the SLT context is limited for the adult population and very limited for the paediatric population. This is illustrated by a systematic review on shared goal setting in adult rehabilitation wherein SLTs and people with communication disorders were underrepresented in the reported studies (Rose et al., 2017), and a recent scoping review on shared goal setting with children with developmental disabilities, in which none of the reported interventions were developed for, or were used in, the SLT context (Ryan et al., 2024). Berg et al. (2017, 2016) explored the experiences of SLTs and adults with aphasia regarding goal setting in rehabilitation centres in Norway. Adults with aphasia experienced collaborative goal setting as vague, sometimes not recalling any goal setting, despite the existence of their personal goals (Berg et al., 2017). SLTs want to place greater emphasis on actively involving people with aphasia in goal setting but express a lack of communication techniques and tools (Berg et al., 2016). Similar work has been done by Hodgetts et al. (2018) with adolescents with autism spectrum disorders, their parents and the involved professionals, among which one SLT. They reported minimal involvement of adolescents in their goal setting which was ascribed to barriers like limited time and service funding, paucity of training and tools and great variability in the language abilities of adolescents (Hodgetts et al., 2018). Gallager et al. (2019) did ask children (10–13 years old) about their ideal school‐based speech and language therapy services and they wished for relevant activities that help them engage with school activities and social relationships.

### Study aim and research question

This study aims to describe common principles that need to be considered for shared goal setting in clinical practice.

We formulated the following research question:

What are the perspectives and needs of speech and language therapists and persons with communication disorders regarding shared goal setting in routine Dutch SLT services?

## METHODS

### Design

This qualitative study was carried out in different settings, being routine speech–language therapy services in practices, primary education and neurological rehabilitation centres throughout the Netherlands. Data collection consisted of videos of goal setting, and reflexive interviews with dyads of SLT and clients, following the principles of video‐reflexive ethnography (VRE) (Idema et al., 2019). Data analysis was based on reflexive thematic analysis (Braun & Clarke, 2021). The study received ethical clearance from the Internal Review Board of HU University of Applied Sciences Utrecht (number 179‐000‐2022). The reporting was guided by the COREQ guideline for reporting qualitative studies (Tong et al., 2007).

### Methodologies

VRE is a qualitative, collaborative methodology that emphasizes the expertise of people who provide and receive day‐to‐day care and who, therefore, are prime candidates to make sense of these complex situations (Idema et al., 2019). We utilized VRE in this study to make the tacit activity of goal setting explicit and to understand and use strengths and weaknesses for designing future practices (Mesman et al., 2019; Morgan et al., 2019). VRE comprises collecting video footage of situations which are deemed meaningful by the researcher and the participants in relation to the research question (video ethnography). Short clips of these videos are selected and reviewed by participants and the researcher during an interview in which they collaboratively reflect on the visual data, which contributes to the trustworthiness of the findings (Idema et al., 2019). The use of video as a visual reference during an interview is expected to be beneficial when interviewing people with receptive and/or expressive language difficulties, who we expected to include in this study (Lyons & McAllister, 2019). We combined VRE with reflexive thematic analysis because we valued the combination of gaining access to the ‘insider’ perspective during data collection and maintaining the ‘outside’ perspective of the researcher during data analysis.

### Setting and participants

Participants were SLT/client dyads. SLTs of all levels of experience were eligible for participation. Children, adolescents and adults with a developmental or acquired language, speech, fluency, voice disorder or hearing loss were eligible for participation. Information flyers and informed consent letters were available at the B1 Dutch language level and with visual support to enable participation of people with language comprehension problems (Council of Europe, 2001). In line with the study aim we recruited the sample from diverse SLT settings to ensure inclusion of a wide range of people with communication disorders. We used a purposive sampling strategy, which was orientated on the age and diagnosis of the clients. We strived to include two dyads in four groups, in each group an SLT and: (1) child aged 4–11 with developmental language disorder or speech sound disorder; (2) adolescent aged 12–17 with developmental language disorder or speech sound disorder; (3) young adult aged 18–25 with speech sound disorder or voice disorder or fluency disorder; and (4) adult aged > 25 with voice disorder or fluency disorder or dysarthria. The diagnoses per group were selected from a list of the most frequently reported communication disorders per age group in Dutch primary SLT care, but we considered that, due to practical limitations, some deviations from these diagnoses could occur (Meijer et al., 2020). We first recruited SLTs inside and outside the authors (LB, RD, KN) networks via email, phone, social media and newsletters. After obtaining informed consent from the SLT, we instructed them to invite eligible clients to participate and they obtained written informed consent from the client. For children under the age of 12 informed consent was obtained from one parent. We succeeded in including six dyads, which led to 12 participants. In one case, the discussion of the client's goals took place with only the parent present, in which case the interview was held with the parent. All participants agreed to participate after reading the information letter and no one dropped out at a later stage. The characteristics of the participants are shown in Table 1.

**TABLE 1:** Characteristics of the dyads of SLT and client.

**SLT ID**	**Gender**	**Setting of the video recording**	**Client ID**	**Age category** ^a^	**Gender**	**Diagnoses**
SLT01	Female	Community practice therapy room	Cl01	Child	Male	Auditory processing difficulties
SLT02	Female	Community practice therapy room	Cl02	Young adult	Male	Stuttering
SLT03	Female	Community practice therapy room	Cl03	Adult	Male	Voice and speech disorder
SLT04	Female	Community practice therapy room	Cl04^b^	Child	Male	Developmental language disorder
SLT05	Female	School‐based therapy room	Cl05	Child	Male	Developmental language disorder
SLT06	Female	Rehabilitation Centre therapy room	Cl06	Adult	Male	Aphasia

*Note*:

^a^ Corresponding ages as described in the setting and participants.

^b^ In this case, the video recording and the interview were conducted with the mother of the client.

### Data collection

Data collection took place in two phases to reflect VRE methodology: (1) video recording of one appointment where therapy planning or goal setting was part of the appointment; and (2) semi‐structured interviews in Dutch, individually conducted with SLTs and clients, during which footage from participants’ own therapy sessions was reviewed. The whole process was pilot‐tested with one SLT/client dyad who did not participate in the study.

### Phase 1: Videorecording

The SLT was instructed to choose a session in which the treatment plan and/or goals were discussed with the client and/or their parent or significant other. All SLTs used their own mobile device to record the session in the usual therapy room. The SLT and client were told to hold the session as usual and received the information that the research aim was to investigate goal setting in general. Prior to the recording, no additional information about (shared) goal setting was provided, and no third party was present during the recording to minimize chances that SLTs adapt their behaviour for the video recording, thereby contributing to trustworthiness. After the appointment, the SLT transferred the recording via a secured data transfer link to researcher LB and deleted the recording from their device. Video recordings lasted between 8 and 50 min.

### Phase 2: Video‐reflexive interviews

Video recordings were viewed in full by author X, who selected three to four clips of 30–90 s for review during the video reflexive interviews. We hypothesized that goal setting is often done implicitly by the SLT and that clients undergo it. Using video footage facilitates the conversation and reflection about these implicit behaviours and prompts participants to voice their perspectives on something they might not have considered before. The video clips were used as a starting point for reflection, rather than wanting to analyse the specific behaviour in every clip. Therefore, clips had to show behaviour relevant to the research question, for example, talking about a treatment or goal or plan, or presenting assessment results together with goals. This selection strategy excluded clips showing SLT intervention activities or scheduling the next appointment. Examples of clips were the discussion of a written treatment plan, numeric rating scale questions related to experienced problems, SLT asking clients or parents directly for their opinion on goals or clients offering ideas for their goals. During the interview, all participants were invited to propose other clips, which none of the participants did.

The interviews were conducted by X at a location of convenience for the participants. This was either at the SLT practice, the home or workplace of the participant or a quiet space in the public library. Except for two SLTs, X did not know the participants prior to the interview. The interviews were informed by an interview guide developed by authors X, X and X and reviewed by the full research team (see Supporting Information). The interview guide consisted of three parts. In the first part, the interviewer established the goal and context of the interview. To create a safe environment, it was pointed out that the review of the footage was not intended to assess the skills or knowledge of participants. The second part focused on the review of the video clips and had the goal of inviting participants to elaborate on the footage (Idema et al., 2019). The questions were based on solution‐focused questioning, containing broad open‐ended questions, for example, ‘What appeals to you in this clip?’, ‘Do you want to tell me more?’; followed by prompts like ‘How was this for you?’, ‘How come you do it like this?’ and ‘Which other factors influence your practice/experience?’. The second part started with reading aloud and showing an adapted definition of shared decision‐making by the Dutch Ministry of Health, Welfare and Sport (2022) followed by an open question: What do you think about this definition? Participants were then asked a scaling question ‘On a scale of 1–10, how shared was the goal setting in the footage applying the definition?’, after which they were asked to elaborate on the strengths they saw in the video and what they think is required to make the goal setting more shared (Mesman et al., 2019). The interview concluded with a miracle question that encouraged participants to imagine a future state in which shared goal setting was perfectly implemented, what that would look like, and what steps were taken to get there (Mesman et al., 2019). The SLT interview guide contained an extra question: Do the domains of the ICF influence the conversation with the client about goals? This question was added as most Dutch SLT guidelines advise to set goals for all ICF domains, being bodily functions, activities, participation, personal factors and external factors. Interviews lasted between 23 and 75 min.

### Researcher characteristics and reflexivity

The first author, X, is trained as an SLT, has 2 years of clinical experience and is currently a PhD candidate. After each interview and during data analysis she engaged in journaling about her personal, disciplinary and topic‐related reflexivity (Braun & Clarke, 2021, p. 13). X and X are trained SLTs, researchers and lecturers in SLT education programmes. They are experienced in qualitative research with communication with vulnerable people and supervised data collection and analysis. X is a physiotherapist and researcher and X is an SLT, researcher and lecturer in clinical health sciences. Authors x, x, x and x have been previously engaged in research on goal setting in allied healthcare and all authors are concerned with the implementation of person‐centred and accessible healthcare services.

### Data analysis

The video recordings were not further analysed. Interviews were audio‐recorded, de‐identified and transcribed verbatim aided by the transcription software Amberscript (Amberscript Global, 2023). Analysis of the interview transcripts took place by engaging in the phases of reflexive thematic analysis which are described in Table 2 to support the transferability of the analysis (Braun & Clarke, 2021). Data analysis started after the tenth interview and continued parallel to the remaining data collection. The analysis was carried out by LB in regular collaboration with RD and KN as a means of investigator triangulation. Coding and data management were assisted using the software ATLAS.ti 23 (Cleverbridge, 2023). During the coding phases RD and KN each coded a sample set of two transcripts and discussed their respective codes with LB, which led to slight adjustments in the coding strategy regarding semantic and latent coding (Braun & Clarke, 2021) LB. In the theme generation and development phases LB regularly discussed the themes with the entire research team as a means of reflecting on the analysis. Theme refinement was done collaboratively between LB, RD and KN.

**TABLE 2:** Overview of the analysis process.

**Phase of reflexive thematic analysis (** **Braun & Clarke,** 2021)	**Actions taken per phase**
Familiarization	LB read all transcripts and took brief notes related to each interview and the whole dataset
Coding	All transcripts were coded inductively by LB. Segments that related to the research questions were identified and tagged with code labels
Generating initial themes	Codes that might share a core concept were clustered into preliminary themes. This phase was done separately for SLT and client interview transcripts. This resulted in a table each for SLT and client interviews, showing candidate themes, subthemes, related codes and sample quotations
Developing and reviewing themes	Preliminary themes from SLT and client interviews were reviewed against the coded interview segments and the whole dataset. This resulted in refined tables and visual thematic maps of themes and subthemes for SLT and client interviews
Refining, defining and naming themes	In a full‐day meeting LB, RD and KN discussed the thematic maps from SLT and client interviews. If applicable and relevant, shared themes were developed. We composed names and definitions for each theme. The product of this phase was a refined thematic map and one collated table containing all themes, subthemes, related codes and sample quotations

## RESULTS

Data analysis resulted in four themes, of which two contain subthemes (Table 3). Initially, we coded client and SLT interviews separately and generated thematic maps for each group. During the theme refinement phase, we found that the perspectives and needs of SLTs and clients overlap and complement each other. We decided that merging the themes would lead to richer and more multifaceted themes, and simultaneously reduce the complexity of two separate sets of results. Nonetheless, some themes were more informed by SLT views, meaning that the topics discussed under the theme were more present in SLT interviews than in client interviews. We indicated this in the description of the themes. Another aspect that arose during the analysis was the distinction between *goal talk* and *goal‐setting process*. Based on the video footage, participants reflected on the actual conversation about goals, and the goal talk. Reflecting on goal talk also led participants to discuss the broader process of goal setting, which was not necessarily captured in the video footage. We use the terms *goal talk* and *goal‐setting process* to refer to this distinction.

**TABLE 3:** Overview of themes, subthemes and short descriptions.

**Theme**	**Subthemes**	**Description**
1. Goal setting is a complex process	1.1 Goals talk serves various purposes	Setting goals is not an *on‐the‐spot action* but rather a *discovery process*, which requires time and space
	1.2 Power and vulnerability	
	1.3 Discovering goals takes time and space	
2. Goal setting needs to be communication accessible	2.1 Communication vulnerability	Necessity and benefits of communication and therapeutic techniques to make goal talk communication friendly
	2.2 Strategies for effective information sharing and collaboration	
3. Communicative participation goals are hard to grasp	None	This theme centres around the difficulty of setting goals regarding the ICF^a^ domain of participation
4. Importance of relationship	None	Therapeutic relationship as a prerequisite and a consequence of shared goal setting

*Source*:

^a^ International Classification of Disability and Health Framework (WHO, 2001).

### Theme 1: Goal setting is a complex process

Both SLT and clients stated that goal setting is not an *on‐the‐spot action* but rather a *discovery process*. The way SLTs explicitly plan and communicate this process varies, and depending on how the SLT do this, it offers opportunities for shared goal setting. An example of an opportunity for shared goal setting is a written form, developed by SLT04, which gives parents the opportunity to think about the therapy goals of their child before talking about goals. SLT04 stated that she does not consistently use the form:

Sending that form in advance […] with some parents I do this and with some parents I don't. Why do I do it sometimes and sometimes I don't? No idea.

SLT01 currently sets the goals and gives the client the opportunity to adjust them:

Of course, people can indicate when they don't agree with the goals, then we can adjust them. But we do not make goals together, we can adjust goals together.

SLT06 said that she explicitly explains the goal‐setting process to her clients and stresses the importance of doing this in a shared way:

Some people come here and just want to practice reading words, and they might find a talk about goals unnecessary. They maybe want to do it quickly, but then they want to just start therapy. It is not always clear for everyone that we first need to be on the same page about the goals, and that is very important. Because then we can practice more focused.

The aspects that make planning and communicating the goal‐setting process complex are elaborated in the subthemes.

#### Subtheme 1.1: Goal setting serves various purposes

This theme was mainly informed by SLT interviews. All SLTs indicated that goal setting combines different purposes which do not necessarily follow a linear pattern but are more likely to be addressed iteratively. Talking about goals is often combined with explaining assessment results and psychoeducation, as SLT04 explains:

I find it important that they know, because this is about her own son, what I saw and what I am doing these last weeks in testing him. Yes, so we know the results and that's a basis to further discuss together.

It is also necessary to discuss aspects of therapy, such as intensity and the need for homework and to build motivation of the client, as SLT06 describes the goal talk after a period of working with a client:

It's good to dwell on this for a moment, because if he thinks: God, it will never get better, then he will not put energy in this. But you do notice he has hope and he sees that he actually improved. But he needs to be reminded of this […] like: ‘This is how you entered here and this is how much you have improved’ so he keeps up his motivation.

Some clients also attributed different purposes to goal setting. Talking about goals provides insight into how they can be achieved, that is what therapy is going to look like. Revisiting goals regularly was valued as a means to reflect on one's progress and to stay motivated, like Cl03 said:

She comes back to the goals regularly, so every time we have a conversation: short evaluation what we have done and what we go on with. I appreciate that.

#### Subtheme 1.2: Power and vulnerability

The subtheme encompasses the views of the participants on perceived power imbalances during goal setting, and how that can lead to feelings of vulnerability. This theme was richly described by all participants.

SLTs and clients discussed the aspect of navigating power or control during the goal‐setting process. SLTs feel confident in taking control of the diagnostic process and in sharing information. However, when seeking input from the client they might feel less comfortable to let go of control and to assume an asking‐and‐listening role. SLT02 reflects on this after viewing video footage in which she offers that working on negative thoughts might be a relevant treatment goal:

This is an interesting piece, because sometimes I find it a thin line in when to steer the conversation. Look, I want to give him insights which I think are quite essential for his progress. But on the other hand, I know that I am steering the conversation with this.

Cl02 offered his perspective when talking about the expert role of the SLT:

Yes, true she is [an expert], but that's why I say: consult on everything! And that's why I liked it that she asked me what I want to achieve instead of telling me what she wants to achieve with me.

Balancing when to take control and actively consult with clients could lead to different goals than SLTs might anticipate based on their performance‐based assessments or experience, and that can feel vulnerable. The SLTs accounts of control were mirrored by the adult clients’ accounts of navigating trust and involvement. On the one hand, the SLT is trusted with organizing the goal‐setting process, for example, the interpretation of test results and the timely scheduling of goal talk and goal evaluation. On the other hand, children, parents and adults alike, wish to be actively involved in goal setting and appreciate SLTs efforts to do so. Extrinsic motivation to be involved can stem from the wish to help the SLT do her work. When asked why she felt it was important for the SLT to ask her opinion about her son's goals, the mother of participant Cl04 replied: ‘I can help, I am happy! Yeah.’ An intrinsic source of motivation can be to improve their own therapy process, like stated by Cl03:

Well, I think, if you let it come from the client, that the SLT gains more information about what is difficult for someone. […] Then she can anticipate on that with the treatment.

Some SLTs experienced balancing of power as difficult because they perceive clients as dependent on them and their professional expertise. This dependency can make the client vulnerable because it might interfere with someone's ability to disagree with suggested goals and treatment options and to freely express their opinions.

As SLT02 put it:

But the difference is that he is in a dependent relationship with me and therefore I am critical [about my behaviour], I think. Because, yeah, he is dependent on me, that's the relationship you are in, so if I say something, would he. […] Yeah, you need to be quite confident to say: I don't want this or I don't see this happening like this.

The SLTs who primarily deliver services to children pointed out that children are in an extra dependent position, because decisions about their healthcare are made by parents, which in turn were perceived to be dependent on the SLTs professional expertise. Additionally, some SLTs expressed doubts if children wish to, and are able to comprehend information about their communication difficulties and goals, which leads them to place the responsibility for goal setting mainly with the parents. These layers of dependency make goal talk with children generally more complex than with adults.

Although this aspect was mainly informed by the SLT interviews, Cl01 did place the power of discussing his goals with his mum and the SLT. He reviewed a video clip of the SLT and his mother discussing the treatment plan which was placed on the table and noted that it felt like he was ‘secretly looking at it’ and that ‘Mum and [SLT] did want to talk about it’.

The adult clients and the parent did not raise the aspect of the clients’ dependency on the SLT.

Another situation where SLTs and clients might feel a power imbalance and vulnerability is when they talk about aspects of communication that are being perceived as ‘weaknesses’. SLTs describe that goals are informed by the experienced problems and concerns of the client, (performance‐based) assessment and the clinical judgement of the SLT. SLTs perceive the discussion of these topics as confronting for the client/ parents. SLTs feel they navigate on a line between eliciting barriers in activities and situations to target in therapy but not appearing as focusing only on the weaknesses of a person's communication. SLT01 demonstrates this balancing act in her reflection on a video clip:

I try to make a little joke in between, even though these conversations are actually quite serious. But I try to keep it a bit light and relaxed, so people don't think: ‘oh, what is wrong with my child, we need speech–language therapy, it must be really grave’ or something similar. But we are just here to help the child, to support.

This aspect was mostly informed by the SLT interviews, although Cl06 expressed that he sometimes feels frustrated when talking about his communicative participation during goal setting. After viewing a video clip, in which where he and the SLT discuss his communication problems with different people, Cl06 said:

I can at most [talk] with [neighbour] and a little bit with my eldest son, but I can't even really communicate with my own sons. Yesterday [neighbour] came to my house and then she talks a bit and until then I hadn't talked all day […] that sucks […] so sometimes I feel like […] how should I say this […] petty.

#### Sub‐theme 1.3: Discovering goals takes time and space

This subtheme emphasizes goal setting as a process and the time and space this requires. Specifically, in the client interviews, it became clear that setting goals is a process. People do not enter the therapy room with goals in their minds, and often, they do not know that goals are going to be set. Knowing the steps of goal setting and being able to prepare was valued by some clients. Clients noted it helpful to have space to process information which enables them to voice their own perspectives on the goals.

Cl03 illustrates this in his reflection on a video clip:

And I think I am quite open, I think, but if you indeed get a question fired at you like ‘These are your goals, what do you think about them? At that moment I really need some space to reflect on it by myself, like: this is what you said, do I agree, or yes, do I see it differently.

Applying communication techniques, as discussed in theme 2, helps to create time and space.

Notably, some SLTs experienced barriers in making the time and space for collaborative goal setting. Treating goal setting as a process was perceived as conflicting with common administration rules, for example, health insurance companies asking for a signed treatment plan after a set number of sessions. Other cited barriers were the extra time to adjust materials, less standardization of the goal‐setting process for employees, possible reimbursement issues and simply not having enough time for a goal conversation. Two SLTs did counter that investing time and space in the goal‐setting process can save time at later point in therapy, which is further discussed in theme 4.

### Theme 2: Goal setting needs to be communication accessible

This theme centres around the communication vulnerability of clients and the subsequent need for a communication‐accessible goal‐setting process. Clients frequently reflected on situations when they felt communication was vulnerable when discussing goals. This was, in some cases, related to their respective communication disorders, but this was not always the case. Clients felt communication was vulnerable when they received information about their assessment and how this relates to their goals. Sometimes, clients felt they did not receive this information, or it was hard to process and retain due to the use of jargon and verbose language, lots of information in a short time, and the fast pace of the conversation. For example, Cl06 said he could not read the SLT's report about his test results:

Client06: Just last week I got a […] look [stands up to grab a folder and show a letter]. This is it.

Interviewer: This is the language test.

Client06: That's it, so I got this last week but I actually didn't look at it because, yeah, because I need to read and then I don't manage.

Client01, who was 7 years old at the time of the interview, was aware that his participation in the discussion of his goals was limited, and he ascribed this to his communication ability and later on to his age as well:

Cl01: I just find talking very difficult.

Interviewer: Okay. But now you are talking quite a lot to me. But then it was a bit difficult?

Cl01: Yes, it was just difficult. Sometimes it is difficult, and sometimes it isn't.

[…]

Interviewer: How come you couldn't follow everything? Was it something [the SLT] did or you mom?

Cl01: Mum […] they are also older than me.

Interviewer: Hmm […] you're right.

Cl01: Yes, and when you are a bit older you also understand more.

A recurring aspect was that all clients were unsure what a treatment goal for speech–language therapy entails and how that relates to their communication problems and treatment options. Client06 indicated that during his goal setting conversation with his SLT he didn't know what goals for SLT entail and followed:

No, I found this totally […] I think, I'm not sure if it's in this conversation, but at some point I said: What goals […].?

The confusion about the purpose of treatment goals was twofold: (1) being uninformed because the SLT did not provide an explanation about treatment goals and (2) not understanding the information or forgetting the information in between sessions due to cognitive impairments.

None of the clients reported on expressing to their SLT when they felt communication was vulnerable or taking other actions. Cl03 illustrates this in his reflection on the SLT reading his treatment goals out loud to him:

Yeah, I easily do this: [nods head]. I do think that these are my goals and the things I want to work on. Especially the breathing, I think. But because it was so much, I often just do this: hmm, hmm [nods head], you know.

Perspectives from SLT interviews support the clients’ experiences that information about communication disorders and assessment results can be hard to understand. Some SLTs expressed uncertainty when discussing goals with people with limited health literacy, limited Dutch language proficiency or complex health and social needs. They had doubts if shared goal setting, is feasible with this population. SLT03 illustrated this with an example of another client of hers:

Can you do this with this population? That's what I wonder. Because [client] would say: ‘No, I'm fine, you are the one who knows best, so we are just gonna do it.’ [And then I ask]: ‘But what do you want?’ And then he might start talking a bit […].

Interviewer: You need to make more of an effort or have some tricks to do this […].

SLT03: Yes, and his understanding of his disease is also very […] because he well […] to give you a bit of background: he has a history of drug and alcohol abuse, the children were placed outside of the home. He is illiterate, they have little money because he can't work anymore. What I see of him is that he wants to make the best of everything.

On the other hand, all SLTs said that they might underestimate a person's ability to participate in goal setting and that they feel an effort should be made to accommodate for communication vulnerability. SLT05 shares her experiences in determining children's therapy goals:

I do see this with children […] often they just do what you tell them to. But maybe they themselves have questions about things, but they won't easily ask them. And when you open up the conversation, you notice that children often do have questions to ask.

Alongside acknowledging the influence of communication vulnerability, clients and SLTs offer solutions to make goal conversations more accessible. SLTs’ and clients’ discussed techniques aimed at effective information sharing and facilitating collaboration. All clients valued receiving accessible information about assessment results, possible goals and therapy options. Clients and SLTs alike promote strategies to facilitate effective information sharing, mainly the use of visual aids, the use of words of the client, the use of plain language and receiving written information to take home. Adjusted written materials are specifically important to the person who was a non‐native Dutch speaker and the person with aphasia. The mother of Cl04 tells why this is important to her and what helps her:

Parents are there to help kids at home. For school, for language growing. So parents need to know each week what children learned. Some people from abroad, some parents can't understand […] that makes it difficult.

Interviewer: That makes it difficult, yes. And how can speech–language therapists help you then to understand better? That it becomes easier.

Client04: Every time give a list on paper, few papers. I am at home, look at everything, read and then I give it to [SLT]. Some Dutch words are difficult, and I do Translate [online translation application].

Although, the children couldn't recall receiving much information about their goals, we noticed during the reflexive interviews that they talked quite detailed about what they do in therapy and what they want to achieve. Lastly, referring specifically to the formulation of goals, clients prefer them to be ‘short and concrete’ (Cl03) and written in their own words.

While effective information‐sharing techniques by themselves can enhance collaboration in goal setting, it should be accompanied by specific techniques to empower clients in goal setting. Some SLTs described the benefits of using preparatory activities as input for the goal conversation: journaling about difficult communication situations in daily life, answering a self‐made questionnaire or completing a patient reported outcome measure. Some SLTs recommended to follow an explicit structure for goal setting and talk this through with the client, for example, at the beginning and/or end of a therapy session. The importance of the SLT asking the right questions and asking follow‐up questions was emphasized by SLTs and clients as a means to collaborate in goal setting. Broad questions like ‘What do you want to achieve in therapy?’ often lead to no answer or a vague answer, while questions like ‘What are situations you find difficult in your daily life?’, followed by ‘Is this a little difficult or very difficult?’ and ‘Do you want us to work on this or first on something else?’ are perceived as more effective.

While also used for effective information sharing, visual aids can have a double function by enhancing collaboration and engagement on a deeper level. SLT06 described the effect of the writing while talking technique as follows:

[…] I think that we still work with pen and paper, often with aphasia clients, just to write down like: Let's see, what questions do you find important? or What do you want to work on? […] And then it's not a case of: I just have a list with the goals, but this this is also your list.

Client03 suggested the use of mind mapping so:

if you have a longer conversation, you can be like: well I jotted down these words that I heard you say. Can we look at this or zoom in on this. So you get a little more depth.

### Theme 3: Communicative participation goals are hard to grasp

This theme stems from SLTs discussing how the ICF domains of bodily, functions, activities and participation influence goal setting. SLTs zoomed in on the participation domain and most SLTs expressed that they find it more difficult to set goals for participation than for the functions and activities domain. SLTs described that a person's participation is difficult to grasp, let alone measure, with the tools available to them. Scaling questions, for example, on a scale from 1 to 10, how much impact your voice problem has on your daily life, are often used to capture participation. Some SLTs struggle to ask effective questions for eliciting concrete participation situations, which subsequently can lead to vague participation goals. Some SLTs also described that they first set goals for functions and activities and that participation goals follow at a later stage. For children, sometimes participation goals are only set when there are obvious behavioural problems connected to communication, based on teacher or parent report, or in older children when therapy focuses on compensation techniques. SLT04 summarizes the above as follows:

And it often stays quite vague like ‘gets along better at school’. Yeah, what does ‘gets along better’ mean? […] It needs to be very specific then, like ‘with this peer he has less squabbles’ or ‘frustrations decreased to once a week’. And sometimes I have this type of goals, then we discuss these things explicitly, but in this case not. I didn't find it necessary to expand on this.

However, one SLT (SLT02) explicitly sets goals for participation and perceives this as a simple and relevant way to set goals:

The client is their own reference point. So I don't have to do the assessing, so to say, but the client does it themselves. So if they say: Well, the impact of stuttering on my daily life is a 7, and I want it to be a 3 […] then I don't have to asses this anymore, because in my experience the client knows very well how much they feel impacted by something.

One client (Cl06) explained that for him it is hard to grasp his communicative participation on a daily basis, whether it concerns difficulties or successes. Together with his memory problems this makes developing and evaluating participation goals very difficult for him. He values regular feedback on his progress via his test results, preferably explained to him in simple language by his therapists.

### Theme 4: Importance of relationship

On the one hand, this theme shows the prerequisite of a good client–SLT relationship to mediate the power imbalance and vulnerabilities described in theme 1. On the other hand, it proposes that shared goal setting can in itself impact the relationship positively.

Generally, SLTs want to exude that they are there to help and that they are open towards a person's accounts about their communication problem, goals and preferences. Cl02 explained how the general attitude of his SLT was influencing his communication ability:

Actually, it [talking] is always going quite well with [SLT], but I think that's because [SLT] has a very open attitude and is just really cheerful and caring.

All SLTs described ways to invest in the therapeutic relationship with their clients. SLTs suggest to openly ask about client's expectations and being aware that these might differ from their own. As SLT06 noted, acknowledging any negative thoughts or emotions that might arise when discussing goals, can also foster therapeutic relationship. After observing herself in the video footage, SLT02 described how she consciously invests in the relationship:

Yes, I always do this. I always check in. Because how someone feels can really influence how the rest of the conversation goes. And it helps you establish a connection. […] this is really important for my therapist–client relationship. So I try to be on top of that.

Some clients confirmed that they want to get to know their SLT and vice versa and that having social talk regularly, contributes to feeling connected. This, in turn, can help to discuss experienced problems in communicative participation. Cl03 described how he connects with his SLT:

Yeah, we connect well. When I enter the room, then it's not like we immediately get to work, but first just have some time to talk about your day before you get to work. Personally, I find that very pleasant.

During the third part of the interview participants reflected upon a definition of shared goal setting and how they relate this to delivering or receiving speech–language therapy. The main aspects named were that a well‐executed shared goal‐setting process can build mutual trust between SLT and client. Cl02 experienced the goal setting with his SLT as very ‘shared’ and described the effect of this:

Yes, because if we do it like this, there is lots of trust towards each other. As a result of this you are more likely to say things.

Cl03 described how shared goal setting can build trust by creating clarity:

And I think if you discuss this together in a good way, that as well the expert as also the patient know very clearly what you are going to do.

Another emphasized benefit was that goals are more likely to be relevant for the client's daily life, which is expected to enhance motivation during therapy sessions and to practice at home. This, in turn, could lead to more effective and even shorter treatment. SLT01 reflected on the effect that shared goal setting could have on goals:

Yes, I think on the participation. For example, that you can make goals more tailored to the home environment. That it becomes easier for people to apply the therapy at home.

Lastly, both the clients and SLTs expressed that shared goal setting makes you feel good. Cl01 watched himself on the video and concluded that he would feel ‘a bit happier’ if he could have participated more in the goal talk because in the video he was ‘looking a bit glum’.

SLT02 concluded:

I can really enjoy a smooth process, when you think: Yes, right, we are both really happy.

## DISCUSSION

This study set out to gain insight into the perspectives of SLTs and clients about goal setting and to describe common principles that need to be considered for shared goal setting in clinical practice. The use of video footage and the inclusion of dyads of SLTs and clients proved challenging, but ultimately gave unique possibilities for rich reflections on goal setting. Looking at the bigger picture of the four themes and their subthemes, we deducted that SLTs and clients perceive goal setting as a process of discovery. The feelings of vulnerability that can arise during this process turned out to be a major theme. Several helpful ingredients to shape a (shared) goal‐setting process were illustrated: communication accessible strategies and materials; time and space; clarity about procedures and roles; and specific attention to the relationship between SLT and client. SLTs discussed the need for conceptual clarity around the ICF domain participation and both clients and SLTs experienced shared goal setting as a way to build mutual trust and motivation.

First, we discuss the notion of goal setting as a process, rather than an on‐the‐spot action, which is illustrated in the theme *Goal setting is a complex process*. Before the actual formulation of goals, preparatory steps are taken and once goals are set, they might be revisited frequently. This is reflected during goal talk by referring to earlier conversations, discussing assessment results, looking at possible treatment options and addressing emotions and motivation. The theme *Discovering goals requires time and space* advocates for granting goal setting, and specifically shared goal setting, sufficient time and space. Time constraint is a frequent clinician concern regarding shared decision‐making and shared goal setting, also outside of speech–language therapy. A meta‐analysis by Veenendaal et al. (2023) showed that well‐designed shared decision‐making interventions, aimed at adult populations, do not necessarily increase consultation length. Nonetheless, persons who are communication vulnerable, and especially children, are underrepresented in healthcare research, and research into shared goal‐setting interventions for these populations is needed to reveal their effects on therapy delivery (Jagoe et al., 2021; O'Halloran et al., 2019; Stans et al., 2018). A clear conceptualization of shared goal setting in SLT as a continuous process, which is connected to all other steps of clinical practice, is needed to facilitate the development and application of shared goal‐setting intervention and tools. This conceptualization of goal setting is underrepresented in clinical guidelines, where it often sits like a one‐time activity between assessment and starting intervention.

Second, we discuss the interpretation of goal setting as a process of *discovery*, which emphasizes the mutual effort that is required from SLTs and clients to elicit, discuss and decide on relevant goals. The themes *Goal setting needs to be communication accessible* and *Discovering goals requires time and space* show that clients often do not enter the first encounter with an SLT expecting to set goals, and they might benefit from clear communication about what to expect from the goal‐setting process (Klatte et al., 2023; Stans et al., 2018). This is in line with earlier findings by Berg et al. (2017) in which clients experienced goal setting as vague. Simultaneously, the SLT enters the process with limited knowledge about the person and their goals. SLTs rely on tools like structured clinical history taking and assessments to discover more about the client and their possible goals. The theme *Communicative participation goals are hard to grasp* suggests that SLTs have insufficient knowledge and tools to elicit a persons perceived communicative participation and subsequently set participation goals. Work by Torrence et al. (2016) and Kwok et al. (2023) revealed most goals do not represent the ICF domain of participation, although SLTs intend to target participation during goal setting and intervention. More conceptual clarity of communicative participation and better availability of valid assessment instruments, preferably client‐reported outcome measures, are needed to facilitate shared goal setting for communicative participation (Baylor & Darling‐White, 2020; Kwok et al., 2023).

The use of VRE in this study led to rich reflections of the participants about a specific element of the goal‐setting process: the goal talk. Elwyn and Vermunt (2020) introduce the term goal talk in their goal‐based shared decision‐making model, which illustrates shared goal setting as an iterative process consisting of three goal talks. For each goal talk, being goal‐team talk, goal‐option talk and goal‐decision talk, corresponding tasks are offered (Elwyn & Vermunt, 2020). Engaging in shared goal talk is intricate and requires specific communication and therapeutic skills from the healthcare professional (Elwyn & Vermunt, 2020; Lenzen et al., 2018). Participants in the current study perceived that engaging in goal talk holds the potential for feelings of vulnerability for all parties involved. One aspect of vulnerability was mainly raised by SLTs in the theme *Power and vulnerability* which pointed to clients’ dependency, especially children's, on the SLTs professional expertise. Lenzen et al. (2018) found similar results in a study of Dutch practice nurses. Boldt (2019) describes this as an asymmetric conceptualization of vulnerability, in which the ‘vulnerable’ client seeks help from the expert healthcare provider. However, healthcare providers experience vulnerability themselves, when they need to deliver bad news or when an empathetic conversation, rather than scientific evidence, is needed to answer questions (Boldt, 2019). SLTs in this study felt highly responsible for delivering quality care and were sometimes unsure if they were overpowering clients’ voices with their expertise. Boldt (2019) argues for a more symmetric view of vulnerability that allows for a shared vulnerability of healthcare providers and clients. The results of this study suggest embracing the discovery element of goal setting. This refers to the attitude of the SLT but could also entail explicitly communicating to the client that goal setting is a discovery process and the different types of expertise each party holds. The SLT being the expert on communication and therapy and the client being the expert on their daily life experiences. Applying this could alleviate the SLT from the role of all knowing expert and thereby facilitate shared goal setting.

A specific case of vulnerability is the communicative vulnerability of many people who seek SLT services. The theme *Goal talk needs to be communication accessible* highlights the importance of adapting the goal talk to a person's communication vulnerability and concrete strategies that SLTs can use are proposed. SLTs are uniquely qualified in facilitating communication by using a wide range of supportive conversation techniques (Blackstone et al., 2011; van Rijssen et al., 2022). We advocate to train SLTs and develop tools to explicitly apply supportive communication techniques during the shared goal‐setting process.

Lastly, the theme *Importance of relationship* implies the potential of the therapeutic relationship to mediate the perceived vulnerabilities, that is, dependencies, discussing bad news and communication vulnerability. The concept of therapeutic relationship, its operationalization and effects on communication outcomes have not been extensively discussed and studied in speech–language therapy research (Hansen et al., 2024). Nonetheless, substantial evidence in psychotherapy research and qualitative work in the SLT context points to the significant positive impact of strong therapeutic relationships on the outcome of interventions (Hansen et al., 2024). In the frequently cited concept of therapeutic alliance by Bordin (1979) collaborative goal setting is seen as a fundamental element of a strong therapeutic alliance (Hansen et al., 2024). The theme *Importance of relationship* offers the possibility that engaging in a communication accessible shared goal‐setting process can itself be an effective resource of relationship building between the SLT and the client. The work by Pinto et al. (2012) supports the use of person‐centred interaction styles by a therapist to enhance the quality of the therapeutic relationship. The role of the therapeutic relationship in shared SLT goal setting, including how to measure and to train it, remains a valuable subject of research (Hansen et al., 2024).

### Strengths and limitations

We consider our intention of including a broad sample of people with communication needs and diverse SLT settings as a strength of this study, as it has been possible to develop overarching and common themes regarding goal setting with this diverse population. Simultaneously, the recruitment of such a diverse sample is time and resources consuming, which led to limitations in the final sample. We failed to sample a gender‐diverse group, as we managed to include only female SLTs and male clients, except for one mother. We did not manage to include adolescents between 12 and 17 years of age. There were also no partners or family members present during the videotaped sessions with the adult clients, which could have influenced the conversations and, subsequently, the reflexive interviews. As a result, the transferability of the results must be treated cautiously. We do believe that the use of VRE and reflexive thematic analysis contributes to the credibility of the reported themes and subthemes. The use of video footage in VRE enables in‐depth discussion of concrete behaviour, which reduces recall bias and it facilitates children and those with more severe expressive communication disorders to express their perspectives and needs (Lyons & McAllister, 2019).

### Implications for clinical practice

The findings of this study provide SLTs with starting points for guiding principles on shared goal setting. We encourage SLTs to view shared goal setting as a process, which needs to be explicitly planned and communicated with clients regardless of their age or communication vulnerability. SLTs have expert knowledge and skills when it comes to supporting communication and we believe that applying these skills during goal talks can enhance shared goal setting and foster a therapeutic relationship. Suggestions of techniques arising from this study are being aware of a person's communication vulnerabilities when it comes to processing information and expressing their needs; take time for goal talk and allow processing time at home, using visual aids; and provide clients with information to take home in an accessible format. Embracing the element of discovery in goal setting can alleviate SLTs from the role of the all‐knowing expert and can open the conversation with the client about what they perceive as meaningful goals for their communicative participation. Nonetheless, SLTs lack resources that facilitate shared goal setting.

### CONCLUSIONS

In order to develop practical interventions for SLTs, shared goal setting needs to be made explicit and embedded in policy and clinical guidelines. The themes reported in this study can be used as tentative starting points for developing shared goal‐setting interventions for SLT practice, under the condition that SLTs and people with communication disorders are continuously involved.

## CONFLICT OF INTEREST STATEMENT

The authors declare no conflicts of interest.

## Supporting information



Supporting Information

Supporting Information

## Data Availability

The data that support the findings of this study are available from the corresponding author, LB, upon request. The data are not publicly available due to containing information that could compromise the privacy of research participants.
