# Supplementary material for: Perspectives on goal setting: Video‐reflexive ethnography with speech–language therapists and clients
Source: Int J Lang Commun Disord. 2024 Dec 5;60(1):e13138. doi: 10.1111/1460-6984.13138 (PMC11619741; doi:10.1111/1460-6984.13138)
Supplement: Supplementary file 1 — Supporting Information [file JLCD-60-0-s001.pdf]

Supplemental material 1: Detailed interviewguide for reflexive interviews with speech-language therapists participants

SECTION	ACTIONS	TOPICS	QUESTIONS AND PROBES
Introduction		Explaining what will happen	The interview has two parts. In the first part, we watch videoclips of the conversation that was recorded. Together we are going to discover what happened in that conversation and why it happened that way.<br><br>In the second part, we will zoom in on a topic (which I will reveal later). I look forward to hearing your experiences and opinions on that topic. We'll also take a brief look into the future. We might also look at the videoclips again.<br><br>There are two rules for the interview:<br>- You are the expert by experience, so you are an expert, and I am a curious listener.<br>- There are no right and wrong answers. Sometimes questions may feel strange to you. That's okay, everyone is different. Above all, indicate if you don’t want to answer a question.
Video-reflection		Background on the recorded session	Can you recall the conversation that was recorded?<br>Can you give a little background about this conversation?<br>- Who is the client?<br>- What preceded this conversation?<br>- Can you tell something about what you typically do before or during this type of conversation?

	Letting participant choose from three video clips		I have chosen three short clips that address treatment goals.<br>[give short summary of each clip]<br>Which clip shall we watch first?
	Playback of the entire clip	Behaviour during the discussion of goals<br><br>Motivation for behaviour	We now watch the whole clip.<br><br>After watching the clip: What does watching this clip evoke in you?
	Replaying subsections	Behaviour during the discussion of goals<br><br>Motivation for behaviour	Are there any bits you want to tell me more about?<br>- What did you do here?<br>- What was this like for you?<br>- How come you did this the way you did?<br>- What other factors play a role in how you engage in the conversation?

Break, 5 minutes

Reflection on Shared goal setting	Reading aloud the definition and showing it to participant on paper	Introduction shared goal setting.	Have you ever heard the term shared goal-setting?<br><br>[Reading aloud the definition and showing it on paper]:<br>Shared goal setting is not a one-off action, but a process.<br>It is a process in which healthcare professional and patient discuss together which treatment goals best suit the patient.<br>This includes all options, pros and cons, preferences and circumstances of the patient.
		Perspectives of SLT on shared goal setting<br><br>----------------------------	What does this evoke in you?<br>What do you think is the outcome of shared goal setting?<br>- Positive outcome/benefits<br>- Negative outcome/disadvantages<br>--

	Drawing ICF model	Facilitators and barriers for shared goal setting<br><br>Influence ICF domains<br><br>----------------------------<br><br>Needs and wishes of SLT regarding shared goal setting.<br><br>Facilitators and barriers for shared goal setting	I'm curious... Thinking back to the video clips, in which ones do you think goals were set together? You may give a mark on a scale of 1-10. A 1 means you don't recognize shared goal setting at all, and 10 means all points of the definition are met.<br><br>You give this a x... Can you talk a bit more about this?<br><br>- What do you do to set goals together?<br>- What makes this work well?<br>- What does the patient do to set goals together?<br>- What makes this work well?<br>- In your opinion, what factors influence shared goal setting together? (In other words: what makes you do it or not?)<br>- Looking at the ICF model [drawing it out on paper] you can set goals for the different domains. Do the different domains influence the conversation about goals?<br><br>--<br><br>Miracle question: We are going to dream for a while. Suppose a miracle happens and we are in the future and we watch the videoclips again. Now you see a 'perfect' shared goal setting conversation, so you give the conversation a 10.

			- What would you see?<br>- What made this happen?<br>- What barriers did you overcome?
Round-up			Room for remarks and feedback from the participant.
