# Supplementary material for: Perspectives on goal setting: Video‐reflexive ethnography with speech–language therapists and clients
Source: Int J Lang Commun Disord. 2024 Dec 5;60(1):e13138. doi: 10.1111/1460-6984.13138 (PMC11619741; doi:10.1111/1460-6984.13138)
Supplement: Supplementary file 2 — Supporting Information [file JLCD-60-0-s002.pdf]

Supplemental material 2: Detailed interviewguide for reflexive interviews with client participants

SECTION	ACTONS	TOPICS	QUESTIONS AND PROBES
Introduction		Explaining what will happen	The interview has two parts. In the first part, we watch videoclips of the conversation that was recorded. Together we are going to discover what happened in that conversation and why it happened that way.<br><br>In the second part, we will zoom in on a topic (which I will reveal later). I look forward to hearing your experiences and opinions on that topic. We'll also take a brief look into the future. We might also look at the videoclips again.<br><br>There are two rules for the interview:<br>- You are the expert by experience, so you are an expert, and I am a curious listener<br>- There are no right and wrong answers. Sometimes questions may feel strange to you. That's okay, everyone is different. Above all, indicate if you don't want to answer a queation.<br>Adapted for child participants:<br>I'm L. I'm a speech therapist, just like [name of child's speech therapist]. But I'm also a researcher. Have you ever heard about what a researcher does?<br>I work at a college, a school for adults. A researcher has a lot of questions. She or he tries to find answers to them. That's what a researcher does. I have questions about speech therapy and together with speech therapists and other children we want to make speech therapy even better.<br><br>-> child may tell about himself, hobbies, school<br><br>In a moment we're going to watch the video that [speech therapist] made. And then we are going to chat together about that clip and about speech therapy.<br><br>There are really only two rules for our chat, and they are still quite fun:

			1. You can say anything you want. I listen to you above all and am very curious about what you want to say.<br>2. If you don't want to say something or don't like a question, you can always say so. You can then say, for example, "I don't want to say anything about this" or just "Stop!"
Video-reflection	Letting participant choose from three video clips		Remember the conversation that was recorded?<br>I have chosen three short clips in which treatment goals are addressed in some way.<br>[give short summary of each clip]<br>Which clip shall we watch first?<br><br>Adaption for child participants:<br>Remember that time you were with [name of child's SLT] and she made a video?<br>What did you guys actually do that time...? [wait]<br><br>I watched that video so I also know a bit about what you did. [Speech therapist] talked about how all those tests had gone that you did.... and she told you what you are going to practise at speech therapy. She talked about the *goals* for speech therapy. Do you know what a goal is?<br>[possibly drawing out a football goal, and other goals, e.g. playing a song on the guitar].<br>There are also goals in speech therapy. You talk about the goals and then write down the goals.<br><br>I chose three piesces from this video.<br>Which piece shall we watch first?

	Playback of the entire clip	Behaviour during the discussion of goals<br><br>Motivation for behaviour	We now watch the whole clip.<br><br>After watching the clip: What does watching this clip evoke in you?<br><br>Adapted for child participants:<br>We will now wacth the piece. Then we will talk together about what happened.<br><br>What did you see in the piece?
	Replaying subsections	Behaviour during the discussion of goals<br><br>Motivation for behaviour	Are there any bits you want to tell me more about?<br>- What did you do here?<br>- How come you did it this way/reacted this way?<br>- What did the SLT do here?<br>- How was this like for you?<br>- What other factors play a role in how you engage in the conversation?

Break 5 minutes

Reflection on Shared goal setting	Reading aloud the definition and showing it to participant on paper	Introduction shared goal setting	[Reading aloud the definition and showing it on paper]:<br>Shared goal setting is not a one-off action.<br>It is a process.<br>Health care provider and patient have a conversation.<br>Together, they decide which treatment goals are best for the patient. They talk about:<br>- which goals are possible<br>- pros and cons<br>- what the patient wants.<br><br>Adaption for child participants:<br>I want to tell you about an idea. That idea is about goals for speech therapy:<br><br>The idea is that the child and SLT can make the goals for speech therapy together. You talk about goals together with the speech therapist and your mother. You tell them what you

			would like to practice. The speech therapist tells you what she can help you with. Then you choose the best goals together.
		Perspectives of person with communication needs on shared goal setting<br><br><br><br><br><br><br><br><br><br>Facilitators and barriers for shared goal setting<br><br><br><br><br><br><br><br><br><br><br><br><br><br><br><br><br><br>----------------------------	What does this evoke in you?<br>What do you think is the outcome of shared goal setting?<br>○ Positive outcome/benefits<br>○ Negative outcome/disadvantages<br>Adaption for child participants:<br>What do you think of this idea?<br>What do you like about this idea?<br>What do you not like about this idea?<br>--<br>I'm curious... Thinking back to the video clips, in which ones do you think goals were set together? You may give a mark on a scale of 1-10. A 1 means you don't recognize shared goal setting at all, and 10 means all points of the definition are met.<br><br>You give this a x... Can you talk a bit more about this?<br><br>- What did the SLT do to set goals together?<br>- Why did this work well for you?<br>- What did you do to set goals together?<br><br>Adaption for child participants:<br>I'm very curious now...<br>Just thinking about the video when you were at x.<br>Who, do you think, made the goals then?<br>[If the child struggels, use Playmobil to recreate the situation sees in the video. Child can then point to the playmobil figures] pointing out the person(s)]<br><br>What did [name of child's SLT] do?<br>What did you do?<br>What did mum do?<br>How do you feel about that?<br>--

		----------------------------<br>Needs and wishes of person with communication needs regarding shared goal setting.<br><br>Facilitators and barriers for shared goal setting	Do you have any other ideas that help to set goals together with the speech therapist?<br>---<br>Miracle question: We are going to dream for a while. Suppose a miracle happens and we are in the future, and we watch the videoclips again. Now you see a 'perfect' shared goal setting conversation, so you give the conversation a 10.<br>○ What would you see?<br>○ What made this happen?<br>○ What barriers did you overcome?<br><br>Adaption for child participants:<br>We are going to dream for a moment. Suppose **you alone** could choose how goals are made. What would you like then? [possibly aid child's answer with with Playmobil].
Round-up			Room for remarks and feedback from the participant.
